# Supplementary material for: A single laccase acts as a key component of environmental sensing in a broad host range fungal pathogen
Source: Commun Biol. 2024 Mar 21;7:348. doi: 10.1038/s42003-024-06034-7 (PMC10957995; doi:10.1038/s42003-024-06034-7)
Supplement: Supplementary file 1 — Supplementary Information [file 42003_2024_6034_MOESM1_ESM.pdf]

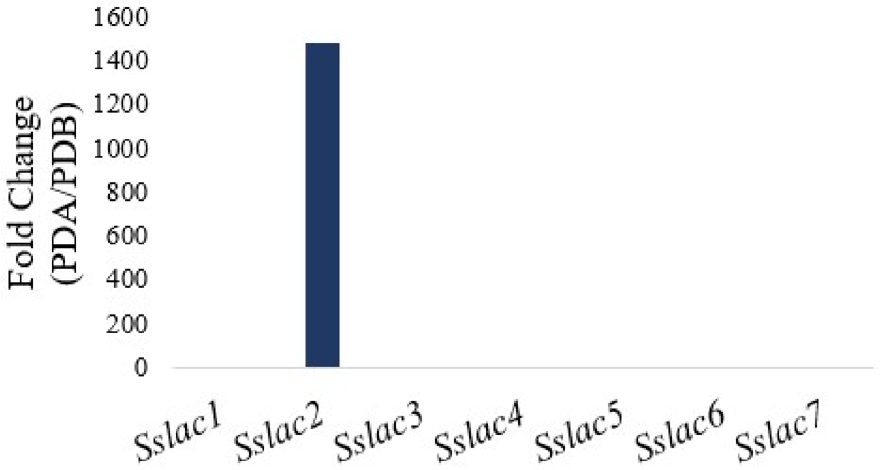

Supplementary Figure 1. Relative transcript fold change of the seven laccases identified in the *S. sclerotiorum* genome when grown on PDA when compared to growth on PDB. Fold change values were taken taken from the transcriptomic analysis performed in Peyraud et al., 2019.

```
SS1G_04196(sslac3)      EIDSGV--
SS1G_05112(sslac4)      EIDSGLRR
SS1G_11927(sslac5)      KTDSGV--
SS1G_00974(sslac2)      QDDSGI--
SS1G_13036(sslac6)      QEDSGI--
                        :  ***:
```

Supplementary Figure 2. Alignment of the C-terminal amino acids from *Sslac2-6.* A conserved DSGx motif is observed at or near the C-terminus of all proteins. *Sslac1* and *Sslac7* are not included as they were missing this motif.

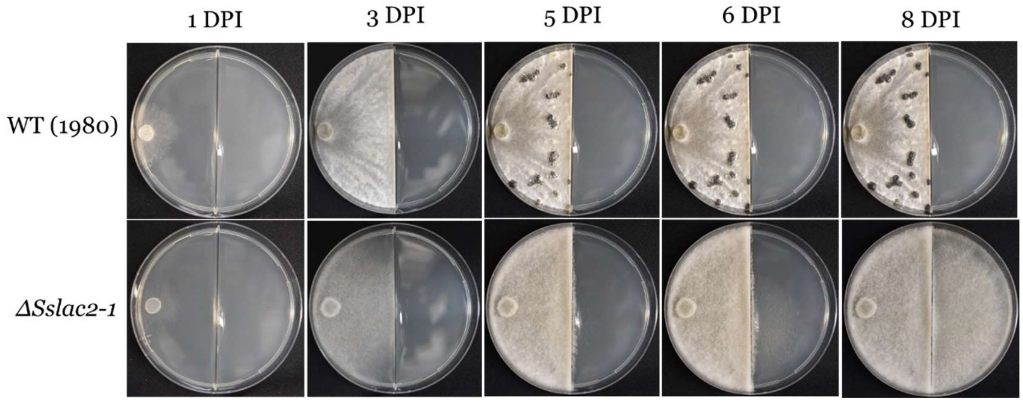

Supplementary Figure 3. Growth of WT (1980) and *ΔSslac2-1* on a PDA split plate over 8 days post inoculation (DPI).

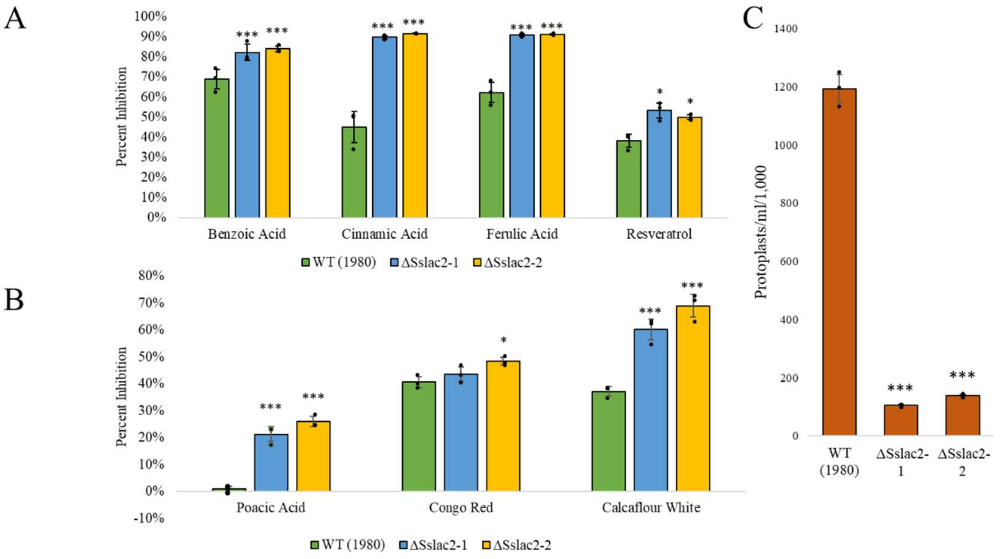

Supplementary Figure 4. Response of the WT and mutants to chemical stresses and cell wall degrading enzymes. A) Relative inhibition of the WT and mutants when grown on plant derived antifungal compounds. Strains were grown on PDA amended with benzoic acid (150 µg/ml), cinnamic acid (150 µg/ml), ferulic acid (500 µg/ml), or resveratrol (200 µg/ml) and compared to growth on a PDA plate amended with DMSO as a control. B) Relative inhibition of the WT and mutants when grown in the presence of cell wall stressors. Strains were grown on PDA amended with poacic acid (50 µg/ml), congo red (150 µg/ml), or calcofluor white (250 µg/ml) and compared to growth on a PDA plate amended with DMSO as a control. C) Protoplast counts from WT and mutants after 3-hour incubation in lysing enzymes from *Trichoderma harzianum.* Statistical analysis utilized a Student's t-test on three biological replicates of each strain (*<0.05, ***<0.001). The source data underlying Supplementary Figure 4B can be found in Supplementary Data 1.

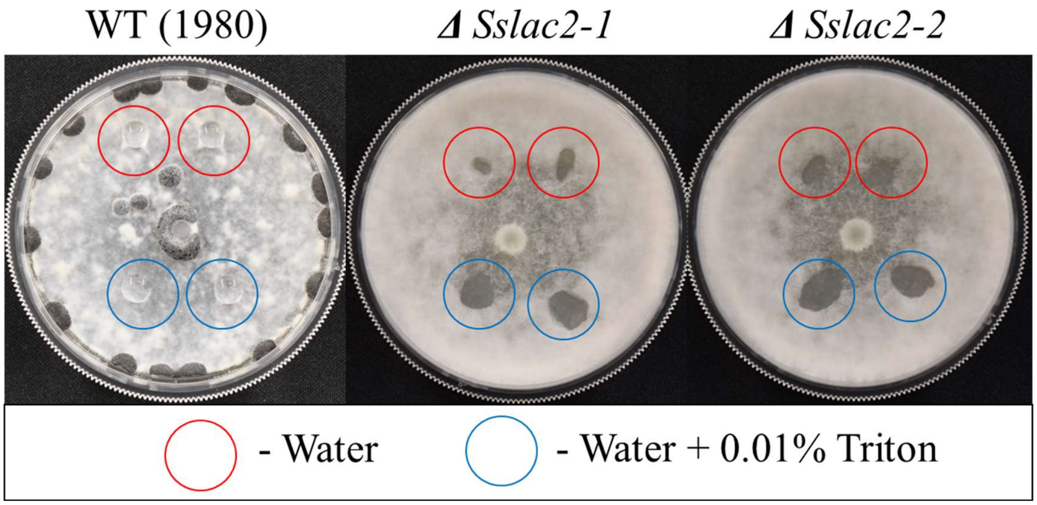

Supplementary Figure 5. Water soaking phenotype of WT and mutant strains of *S. sclerotiorum*. 100 ul of water or water + 0.01% Triton were added to 2-week-old colonies of WT and mutant strains. Photos were taken immediately after droplets were placed.

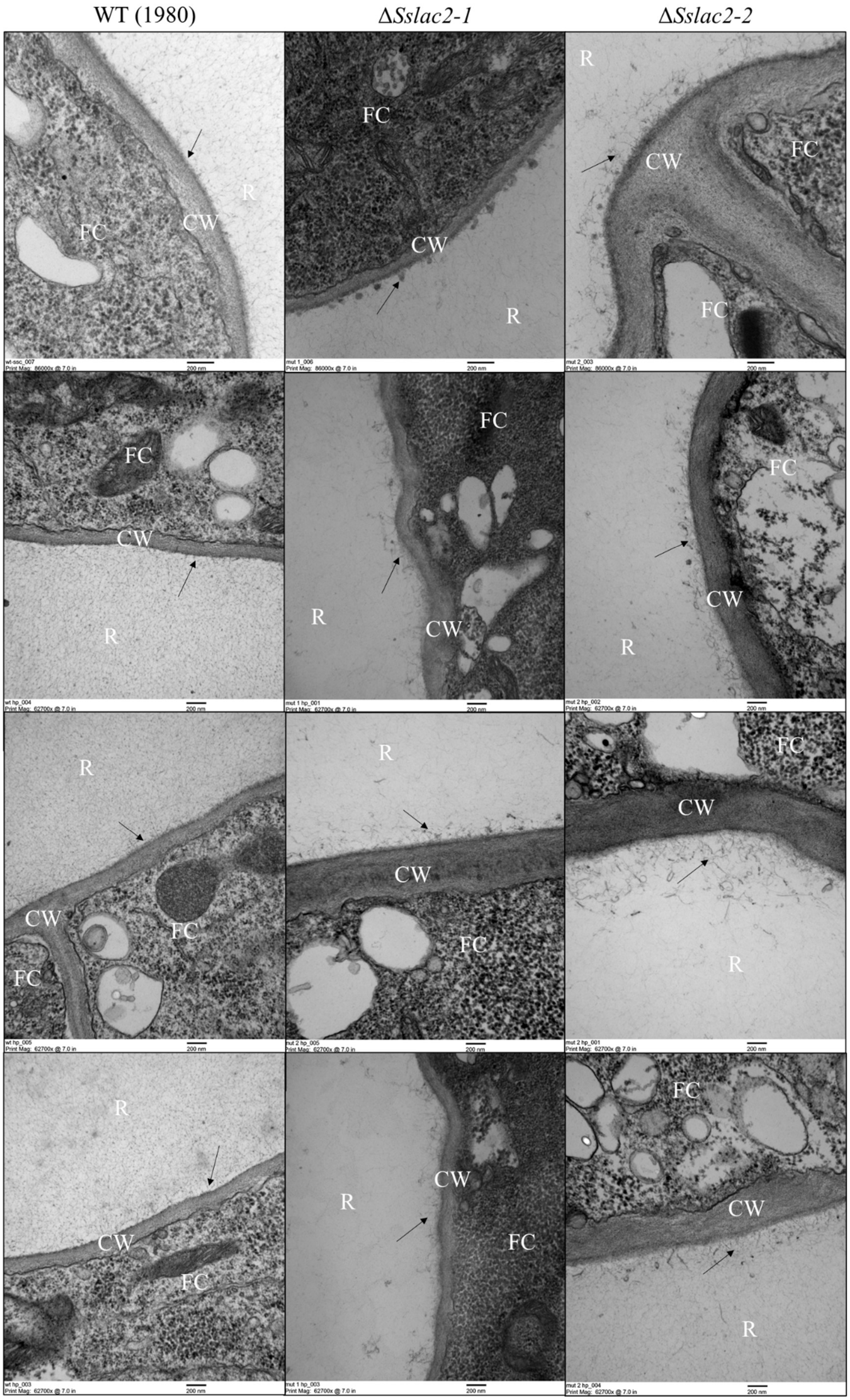
WT (1980)
ΔSslac2-1
ΔSslac2-2
FC
CW
R

Supplementary Figure 6. Transmission electron microscopy (TEM) photos of the cell walls and extracellular matrices of WT (1980), *ΔSslac2-1*, and *ΔSslac2-2*. R – Resin, FC – Fungal Cell, CW – Cell Wall, arrows denote the fungal extracellular matrix.

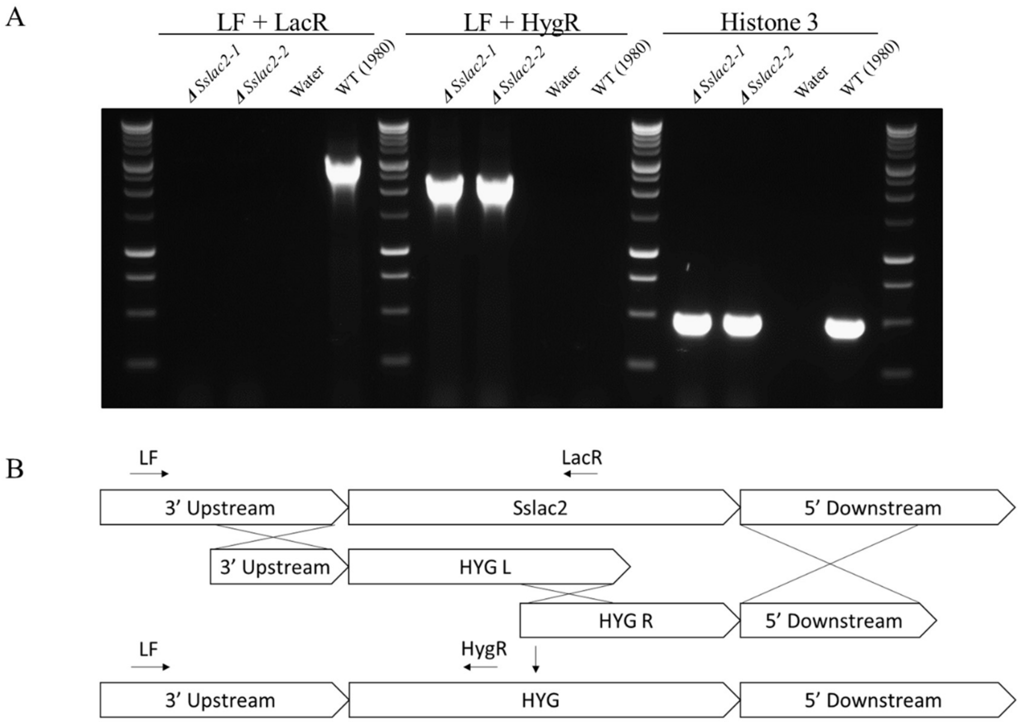

Supplementary Figure 7. Validation of *Sslac2* gene knockouts in *S. sclerotiorum* by PCR. A) Left: PCR with external (LF) and internal (LacR) *Sslac2* primers, Middle: PCR with an external *Sslac2* primer (LF) and an internal (HygR) hygromycin resistance cassete (HYG) primer, Right: PCR of *S. sclerotiorum* histone 3 gene. B) Schematic diagram of split marker gene replacement used to generate *Sslac2* knockout strains. Approximate placement of validation primers are included.

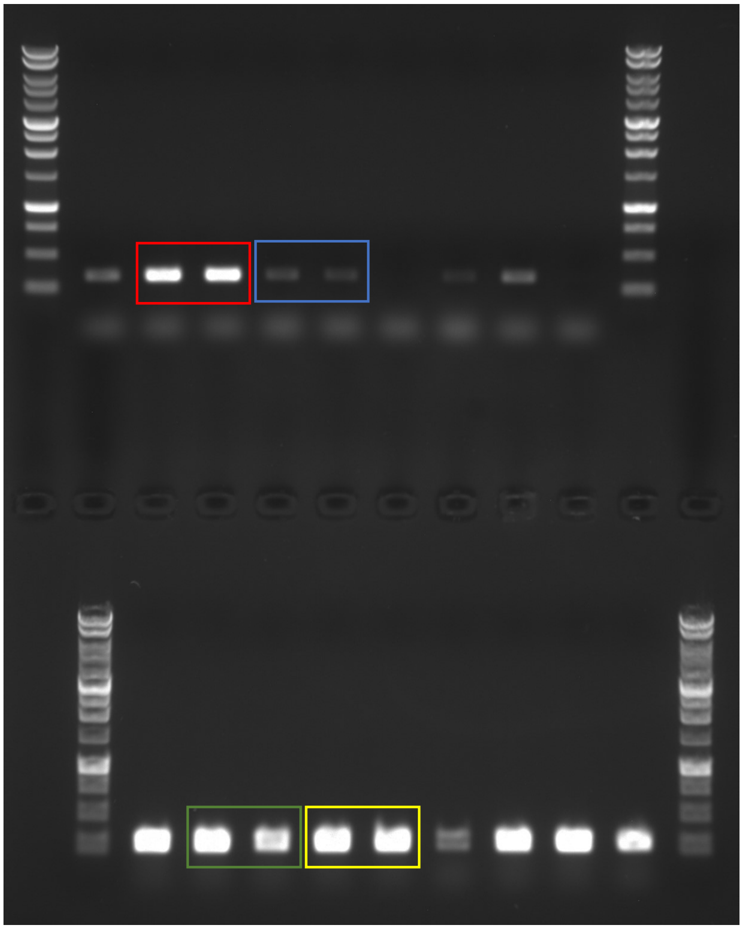

Supplementary Figure 8. Raw Gel of semiquantitative RT-PCR shown in Figure 1c.

Red box – *Sslac2* expression on PDA. Blue box - *Sslac2* expression on PDB. Green box – Histone 3 expression on PDA. Yellow box - Histone 3 expression on PDA.

Ladder is NEB 1 kb Plus DNA Ladder (#N3200S).

Supplementary Table 1. Primers used in this study.

Primer Name	Primer Sequence
Sslac2-LF-F	AGTGGGACATACCTGTGGTT
Sslac2-LF-R	tctaaacaagtgtacctgtgAAGGGACTGTGTTCACCAGA
Sslac2 -RF-F	aatccaatgcgtctagagggCTATCGATCGGTTAAGCTGGT
Sslac2-RF-R	TATCACCGTCACACATCACG
PstI-VIGS-F	aactgcagacggtaagagtgtgaccatc

BamHI-VIGS-R	aaggatcctggagtcatgttcttgggag
Sslac2 Det F	aattcgaggcggttttcg
Sslac2 Det R	atagcttccgacatccatgg
