# Supplementary material for: A single laccase acts as a key component of environmental sensing in a broad host range fungal pathogen
Source: Commun Biol. 2024 Mar 21;7:348. doi: 10.1038/s42003-024-06034-7 (PMC10957995; doi:10.1038/s42003-024-06034-7)
Supplement: Supplementary file 2 — Description of Additional Supplementary Files [file 42003_2024_6034_MOESM2_ESM.pdf]

Description of Additional Supplementary Files

File name: Supplementary Data 1

Description: Differential gene expression values comparing WT (1980) and ΔSslac2-1 from RNASeq analysis.
